# Mitral Valve Entrapment of Preshaped Guidewire During TAVI

**DOI:** 10.1016/j.jaccas.2026.108683

**Published:** 2026-06-03

**Authors:** Barbara Parrella, Cristina Barbero, Federico Conrotto, Alessandro Vairo, Stefano Salizzoni

**Affiliations:** a Division of Cardiac Surgery, Città Della Salute e della Scienza Hospital, University of Turin, Turin, Italy; b Division of Cardiology, Città Della Salute e della Scienza Hospital, University of Turin, Turin, Italy; c Department of Surgical Sciences, University of Torino, Turin, Italy

**Keywords:** anterior mitral leaflet (AML), bailout, minimally invasive, mitral injury, mitral regurgitation (MR), rescue, transcatheter aortic valve implantation (TAVI), transcatheter aortic valve replacement (TAVR)

## Abstract

**Case Summary:**

We report a case of acute mitral regurgitation caused by a stiff guidewire entrapment in the subvalvular apparatus during transcatheter aortic valve implantation. Early detection by the multidisciplinary Heart Team enabled prompt conversion to successful minimally invasive surgery for mitral repair.

**Take-Home Message:**

Mitral injury due to guidewire entrapment is a rare, life-threatening transcatheter aortic valve implantation complication. Key to success is the early recognition of pathognomonic echocardiographic features and immediate surgical bailout. Minimally invasive surgery is a safe, effective rescue strategy. Maintaining a hybrid environment with a scrubbed cardiac surgeon is essential to optimize outcomes in such high-risk, unexpected scenarios.

## Graphical Abstract

**Figure:**
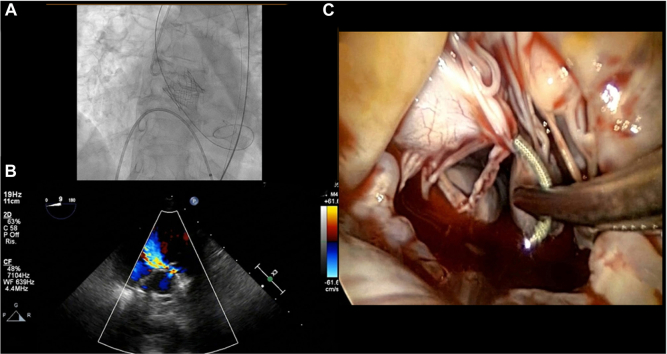

## Clinical Vignette

An 83-year-old woman with several comorbidities was admitted for symptomatic severe aortic stenosis with indication to treatment with transcatheter aortic valve implantation (TAVI).

**Figure 1:**
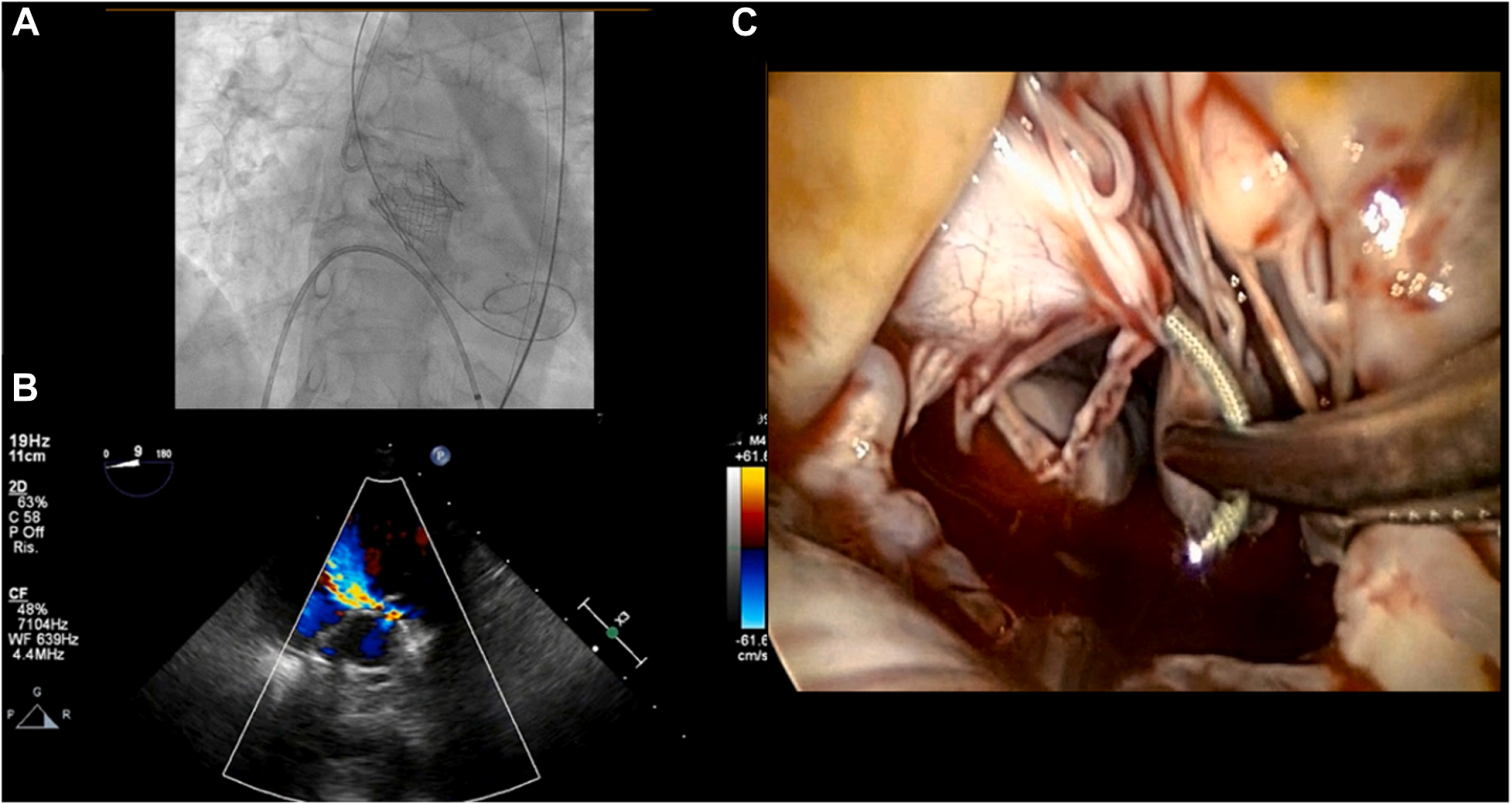
Fluoroscopic, Echocardiographic, and Intraoperative Findings of Mitral Valve Guidewire Entrapment (A) Fluoroscopic check of the positioning result of a 23-mm Sapien 3 Ultra valve (Edwards Lifesciences); evidence of anomalous movement of the Safari guidewire. (B) Transesophageal echocardiography imaging of highly eccentric mitral regurgitation jet caused by anterior mitral regurgitation jet caused by anterior mitral leaflet retraction. (C) Intraoperative endoscopic visualization of Safari guidewire entrapment within the mitral subvalvular apparatus.

The procedure was performed in a hybrid operating room under local anesthesia and conscious sedation. The aortic valve was crossed retrogradely at first with a J-tip wire via an AL-1 catheter and then with an extra-stiff Safari guidewire (XS, Boston Scientific) without advancing the wire beyond the catheter tip to direct the system toward the left ventricular apex, as usual. C-arm right anterior oblique view confirmed the right positioning. A 23-mm Sapien 3 Ultra valve (Edwards Lifesciences) was successfully implanted. Resistance was encountered during attempted removal of the Safari. Repeated withdrawal maneuvers were associated with sudden hemodynamic instability and oxygen desaturation. Transthoracic echocardiography demonstrated acute severe mitral regurgitation occurring during guidewire traction. Multiple attempts to disengage the guidewire using different catheters (pigtail, AL1, multipurpose) were unsuccessful. After induction of general anesthesia and intubation, transesophageal echocardiography showed verticalization of the anterior mitral leaflet during guidewire traction, resulting in massive mitral regurgitation. The procedure was therefore converted to open heart surgery via minimally invasive video-assisted right mini-thoracotomy. Endoscopic transvalvular inspection revealed the tip of the Safari firmly entrapped in the mid-portion of the anterior leaflet (Figure 1). The guidewire was pulled back and cut distally allowing removal through the femoral access. Due to leaflet injury and a residual prolapse, a prophylactic expanded polytetrafluorethylene neochord was implanted on A2 (Video 1). The patient necessitated inotropic support for 48 hours and 6 days in the intensive care unit. Predischarge transthoracic echocardiography showed normal left ventricular ejection fraction, mean gradient of 13 mm Hg on the TAVI without paravalvular leak, and mild proto-systolic mitral regurgitation.

## Discussion

The utilization of preshaped stiff guidewires within the left ventricle during TAVI carries an inherent risk of iatrogenic mitral valve injury. Specifically, guidewire entrapment within the mitral subvalvular apparatus may lead to severe mechanical complications, such as chordal rupture and leaflet trauma. Although these occurrences are statistically rare, they can precipitate acute hemodynamic instability, necessitating an immediate conversion to conventional surgery. Consequently, the presence of a “scrubbed-in” cardiac surgeon, involved at every stage of the procedure, is mandatory to ensure a rapid surgical bailout, which is often decisive in modifying the clinical outcome when such complications arise.^1^

In this context, minimally invasive surgery plays a crucial role. Minimally invasive surgery provides optimal visualization and facilitates precise valve assessment and repair, even in the presence of a newly implanted TAVI. In high-volume centers, this approach can be safely adopted even in emergency bailout scenarios and for high-risk older patients, allowing for rapid intervention while significantly limiting surgical trauma.^2^

These findings further emphasize the pivotal importance of a dedicated Heart Team approach.

## Funding Support and Author Disclosures

The authors have reported that they have no relationships relevant to the contents of this paper to disclose.

## Appendix

Video 1 Post-TAVI Fluoroscopy Showing Abnormal Safari XS Guidewire Motion and Entrapment

Video 2 TEE Evidence of Severe Eccentric Mitral Regurgitation During Attempted Safari Guidewire Removal

Video 3 Intraoperative Endoscopic View of Guidewire Removal and Mitral Valve Repair with PTFE Neochord Implantation on the Anterior Leaflet

Video 4 TTE Result of Mitral Repair; TTE Evidence of Post Operative Residual Mild Mitral Regurgitation
